# Supplementary material for: The effect of odor enrichment on olfactory acuity: Olfactometric testing in mice using two mirror-molecular pairs
Source: PLoS One. 2020 Jul 30;15(7):e0233250. doi: 10.1371/journal.pone.0233250 (PMC7392274; doi:10.1371/journal.pone.0233250)
Supplement: Attachment — Submitted filename: Response to Reviewers.docx [file pone.0233250.s001.docx]

*Biology Department*

*Copley Science Bld*

*304 Caroline St*

*Ashland, VA 23005*

*Ph. 804-752-3147*

*Email: dcoppola@rmc.edu*

*Copley Science Bld.*

*304 Caroline St*

*Ashland, VA 23005*

*Ph. 804-752-3147*

*Email: dcoppola@rmc.edu*

*Copley Science Bld.*

*304 Caroline St*

*Ashland, VA 23005*

*Ph. 804-752-3147*

*Email: dcoppola@rmc.edu*

June 29, 2020

Andreas T. Schaefer, Ph.D.
Academic Editor
PLOS ONE

**Re:** “PONE-D-20-12663
The effect of odor enrichment on olfactory acuity:  Olfactometric testing in mice using two mirror-molecular pairs. PLOS ONE

Dear Dr. Schaefer:

Please find attached our revised manuscript (identified above) which we would like reconsidered for publication in PLoS One. The original work has been thoroughly edited and expanded in response to the reviewer’s comment. My co-author and I feel our manuscript has been significantly improved owing to the reviewers thorough and authoritative reviews and hope you will agree.

For your convenience we have provided below a point-by-point response to each reviewer’s comments. The most significant changes to the manuscript involve the addition of two new sub-headings dealing with “statistics” (pooling in particular) and “control procedures” the latter subsection explaining how we minimized potentially confounding, extraneous odor cues. We also provide a detailed argument below for why vapor-phase concentration measurements, suggested by Reviewer 1 would be impractical.

The track-changes version and “neat” version of our manuscript, as well as original and revised figures, have been loaded through the author portal.

We look forward to your response.

Sincerely,

David Coppola, Ph.D.

Professor of Biology

**Reviewer #1:**

Review of PONE-D-20-12663 “The effect of odor enrichment on olfactory acuity: Olfactometric testing in mice using two mirror-molecular pairs.”

This manuscript sets out to test the ability of unsupervised learning through a process known as induction to improve detection and discrimination thresholds of enantiomeric pairs of odors. Throughout the study mice report the presence of odors using a standard go/no-go behavioral task. As part of this task the authors use an interesting experimental approach for their discrimination assay. Here they ask mice to discriminate a pure odor enantiomer and another that has been diluted with the enantiomer of opposing chirality. Although the experiments in this manuscript fail to find evidence for induction, the authors report detection and discrimination thresholds that exceed many prior reports.

I have two major concerns that I will also expand on below. First, the justification for pooling data in many instances is not well described. In the least, I hope that the authors will provide statistical analysis accompanying data prior to pooling. Second, a technical concern, the authors claim to report the among lowest threshold measurements ever recorded for any species. While I agree that the data do indeed look promising, several important technical controls including vapor phase odorant measurements are needed to support such a claim. Below I provide a point-by-point critique:

1) The choice of enantiomers seems justified but is somewhat curious given that enantiomers of both carvone and limonene are perceptually dissimilar, therefore it is not clear whether there is anything special about enantiomeric pairs, with regard to odor discrimination. In the discussion the authors point out that enantiomers interact with chiral-specialist and -generalist receptors; however, this is also true for any odors that activate overlapping patterns of OSNs - of which there are many. I recommend a general deemphasis regarding the special case of enantiomers. However, one key advantage that might be mentioned is that the vapor pressure should be nearly the same between enantiomers.

Related to the above point in Line 516: “We also wanted to use enantiomeric pairs because it was assumed that mixture discrimination would be highly difficult” – This may be a false assumption especially given that it is well documented that carvone enantiomers are perceptually dissimilar and activate distinct (yet overlapping) populations of ORNs.

We appreciate Dr. Zak’s point of view on the issue of enantiomers however the key advantage that he mentions in his critique was the first of our multipart justification for using limonene and carvone. See below from p. 6 of the revised MS:

“First, enantiomers have identical physical properties, other than chirality, assuring that discrimination cannot be based on concentration or other properties unrelated to odor quality. Second, as noted above, psychophysical measurements of these compounds have already been reported for CD-1 mice [12] based on data obtained from nearly identical olfactometric equipment (Knosys, Tampa, FL, USA). Third, though limonene and carvone are structurally similar, previous studies have shown that discrimination of limonene enantiomers but not carvone enantiomers can be induced in rodents through passive enrichment [13]. Fourth, we assumed that one of the tasks we chose, discrimination of a mixture of enantiomeric isomers from a pure isomer, would be very difficult, thus leading to rapid threshold determination requiring a minimum of odor exposure.”

As for the rest, only our fourth justification relates to chirality and on this point we admit discrimination difficulty was an assumption that we later disprove. Thus, we are at a loss as to how we might further “de-emphasize” the special case of enantiomers in the manuscript.

2) My primary concern regarding statistics is the justification for pooling data that used different experimental conditions. My main issue is that the treatment between the two groups is fundamentally different and therefore it may not be appropriate to group data together. I request that the authors provide a more detailed description as to their justification. This should extend beyond a lack of effect in either group alone. A formal power analysis might be considered here.

Related to the above point in Line 311: While I do not have a strong objection to the authors pooling the data from the two experiments and allowing readers to draw their own conclusions, I do think that it is necessary to present statistics for each the datasets prior to pooling. In essence these are two fundamentally different experiments (passive odor exposure vs exercise), therefore, at face, it is not intuitive why these data should be pooled.

In response to this critique, we have added a **new sub-section** entitled “Statistics” (p. 6) in which we clarify our use of pooling subgroups for added statistical power. Dr. Zak’s objection to pooling data is clear. Less apparent is what statistical canon he believes we have transgressed by pooling.

At the risk of pedantry, we assert that pooling is a common practice in statistics. Perhaps the most familiar case is the pooling of sample variances in a Student’s t-test to find the significance of the difference between two sample means. In this case, the null hypothesis is that the means of two populations are equal. An investigator rarely knows the population variances but typically we assume that they are equal. Here we need an estimate of the variance that the two populations have in common so we **pool** the two samples to obtain a more accurate estimate of the population variance.

Similarly, we pooled in this study, as we make clear in the revised manuscript, to increase statistical power. In almost every case this proceeds **after** the subgroups (enriched and control) have been shown **not** to be statistically different. Under this condition, our conclusion must be that the subpopulations could have been drawn from the same underlying distribution (and thus can be pooled); that is the meaning of a failure to reject the null hypothesis.

With regard to Dr. Zak’s comment about “power analysis” we read up on power measurements of Mann-Whitney and Wilcoxon tests, (Shiel, G., Jan, S., Randles, R. On power and sample size determinations for the Wilcoxon–Mann–Whitney test. Journal of Nonparametric Statistics Vol. 18, No. 1, January 2006, 33–43) but were uncomfortable with the assumptions and the utility of this analysis in our case, especially *a posteriori (*though we certainly see the merits for using power analysis for determining sample sizes *a prior)*. Furthermore, we consulted our colleague, Dr. Cole Barton, who taught statistics for 40 years at Davidson College, before his recent retirement. He pointed out that 95% confidence limits of the medians would be more useful than power calculations (and more typical). Therefore, we have added CIs to the appropriate figures in response to Dr. Zak’s suggestion.

We believe the statistical part of the manuscript and our handle on the data were improved by Dr. Zak’s comment.

3) Figure 1: please provide an x-axis in these plots.

The X-axis is explained in the first line of the figure legend and could actually add confusion if reduced to a word or phrase for the graph. Therefore, we have chosen not to make this change for the sake of clarity.

4) Figure 2: These plots might be more effective if the data for each mouse is plotted on the same axes for each of the three conditions. Also related to these data, were the mice only tested once at each odor concentration, or is the data at each point an average of several sessions? If so, please provide error bars.

All of the graphs have the same axes. Thus, “the data for each mouse is (are) plotted on the same axes for each of the three conditions.”

The mice do not get multiple threshold tests within a treatment group (graph) and thus there are no error bars. Such replication is exceedingly rare in the olfactory psychophysical literature for obvious reasons. Our mouse threshold measures, however, were based on an unprecedented (as far as we know) amount of testing! For convenience, we include the part of the methods below where this is described:

**“In order to pass at a particular dilution, a subject had to achieve a minimum of 85% 184 correct in the last two blocks of a session or to have a minimum average of 85% correct for the 185 last 3 blocks. If a subject failed to reach criterion in the first five-block session, she was given a 186 second five-block session. If the subject failed again, she was given a third and final-seven block 10 session to meet the 85% criterion.”**

Thus, each of the threshold measurements was based on 340 individual tests over 12 blocks and 3 sessions.

5) Figure 3: It is very hard to follow where the n values come from throughout this figure. Please revise the text to add clarity and justification when data was pooled.

Text has been added on p. 18 and 19 to clarify the sample sizes used and, as in the original manuscript, every data point is shown.

Figure 3: I am also generally confused about the message of the last panel in part A. If detection occurs at significantly lower thresholds than discrimination after enrichment, are these values indistinguishable prior to enrichment? Some other measures might be informative for the interpretation of this data, for example, plotting the change in threshold for discrimination/detection with exercise/enrichment.

The second panel from the left in Figure 3 A shows there is not a consistent effect on thresholds of passive enrichment in the pre/post design. And there was no consistent effect of exercise (left panel) on threshold though the sample sizes are small (typical of other studies). Given this lack of consistent effect of enrichment we pooled these groups to determine if discrimination in the pre-post tests had different thresholds than for detection. Subsequently, when we pool **all** limonene data we don’t find any significant difference between detection and discrimination thresholds. While this final test superseding the results of Figure 3 A, we decided to leave the last panel in part A for completeness.

6) Major technical concern: The dilutions used are very small and may be impacted by the viscosity of mineral oil when transferring between serial dilutions. Did the authors use a device such as a photoionization detector to measure vapor phase odor concentrations? Without such measurements to control for dilution-to-dilution variability, it is hard to be certain that the authors are truly delivering the odor concentrations that they calculate. Furthermore, it appears that the claim of the low detection/discrimination thresholds are based on data from a number of mice, but it unclear whether these were single blocks of trials in each animal or if the low thresholds could be reliably measured across several days/sessions of behavior. Can the authors please explain if these data are from single blocks of trials or are averages of several sessions.

**Dilutions**

We agree with Dr. Zak that it is unlikely our serial dilutions are highly accurate since they were volumetric measurements done by hand with a viscous solvent. However, a key advantage of serial dilution is that the experimenter is measuring milliliter quantities not microliter quantities. Moreover, any errors in measurement are merely additive across dilution steps! Consider two simple simulations we performed to illustrate this fact. In the left graph (below), we simulated 19 serial dilutions steps and used a random choice generated to choose one of seven measurement errors (-15, -10, -5, 0, +5, +10 or +15 %) on **each** dilution step. Note that at end of 19 steps the average accumulate error was + or - 26.7% (range 10.3% to 37.5%). In the second simulation shown in the right figure (above) we considered systematic loss under the assumption that with viscous liquids volume measurements would typically be underestimates. The simulation considered average loss of liquid during measurement of 2%, 5% and 8% on each step of 19 dilutions. Note that even with 8% systematic loss the accuracy of the actual, final dilution is less than a log-unit (312%) different than the nominal concentration. It is also worth considering that our dilutions were remixed every two weeks or more frequently if we had any suspicion of contamination. Thus, dilution series were remade scores of times during the 2 years of our study. Given this amount of repetition, it seems likely that measurement errors in one direction would be cancelled out by errors in the other direction over the duration of the study.

Finally, none of our conclusions of our study would be changed by even the extreme cases that were found in our simulated levels of inaccuracy. We make some of these point in new text found on p. 7 of the revised manuscript.

**Vapor Phase Concentrations**

In the revised manuscript we have not measured vapor phase concentrations as requested by Dr. Zak, only estimated them from Henry’s Law calculation. We hope the following rationales will convince Dr. Zak to accept our results without these measurements:

1. There is no analytical method that we are aware of, including photoionization detection, which could measure the lowest concentrations used in this study. Sato et al, 2015 measured the accuracy of their 100-fold dilution series by measuring the dose-dependency of the peak areas of a diluted (R)- (− )-carvone solution using a gas chromatography-mass spectrometry (GC-MS). On an encouraging note, related to Dr. Zak’s comment about measuring (above), they found that manually performed serial dilutions were surprisingly accurate even with a viscous solvent (they used di-propylene glycol). However, the sensitivity limit of the GC-MS was in the 10 PPB range, well above our lowest concentrations that were in the parts per quadrillion range!

2. We are quite confident in stating that fewer than 1% of previously published olfactometric studies have included measurements of the vapor-phase concentrations of stimuli. Indeed, because of the difficulty of calculating head-space odor-concentrations the vast majority of previous investigators---including most of the of authors we referenced in our citation list---only report liquid-phase concentrations. Thus, it seems patently unfair to hold us to such a stringent standard.

3. As noted in our response concerning measurements of viscous liquids, even maximum possible differences between the nominal and actual concentration of stimulus dilution steps would have a *de minimis* effect on our two main conclusions: (1) there is no effect of limonene and carvone induction/enrichment on olfactory thresholds in mice; And, (2) when given sufficient practice, mice have olfactory thresholds, at least for limonene and carvone, that are far lower than previously appreciated.

4. Valid quantitative measurements of even our higher odor concentration steps are beyond the capabilities of our largely behavioral laboratory. Thus, in order to get such measurements we would have to incur significant costs in a fee-for-service situation as collaborators would be hard to find for such a pedestrian chemical question. Given limited financial resources, we felt our money could be better spent elsewhere particularly since our lowest concentrations are beyond the sensitivity of normal laboratory instruments..

**Replication**

A claim that we try to back up with our literature review is that ours is among the most replicated study of mouse thresholds in existence. The average mouse in our limonene tests underwent nearly 2,000 separate trials to reach threshold, sometimes taking weeks to achieve (see Figure 1B). In the end, we measured thresholds for 36 mice and replicated some of those thresholds on the same mouse in the pre and post-tests.

As noted in the excerpt on threshold criterion (above) a mouse was allowed up to seventeen 20-trial blocks to pass criterion on a single odor. Previous studies have typically used a single 100-trial session to determine threshold (see Laska references in our literature cited).

As should be clear from our graphs, “super-sensitivity” which we will define here as a part per quadrillion threshold, was not a rare finding in our study. In fact, most of the mice in the limonene group reached this threshold or better. Thus, we find extremely low thresholds for limonene discrimination and detection and we show these can be replicated in most mice provided they are given sufficient practice AND are sufficiently motivated.

7) Line 331: Can it really said that the null-hypothesis was rejected for the pre/post experiments given that statistical power is very low with n = 3 and 4? This is especially true for the passive enrichment group given the large variance across replicates.

We can’t find the passage in question at Line 331 and searching the document for “null”, or “null-hypothesis” or “hypothesis” does not return a similar statement. We agree with the Dr. Zak that if the sample size is very small a rejection of the null is far more likely (actually its inevitable with non-parametric tests of the sort we used) and far less meaningful than if the sample sizes were large.

8) Perhaps the authors can explain why the detection/discrimination curves all seem to fall to chance (50% accuracy) at a specific dilution of the odorant. A reasonable assumption is that there should be some performance degradation that appears as the animals begin to approach detection threshold. Often times data such as these are plotted as a psychometric function that can be fit with a hill equation. In this case, it appears the data would be better approximated by a step function. Can the authors please provide some insight as to why performance drops off all at once?

**Threshold measures for CD-1 mice from Joshi et al., 2009 (see references cited). Like the current study these authors used a descending method of limits albeit with a more rigid criterion threshold which leads to lower thresholds. Note the precipitous drop from high level performance to chance performance typically from one odor concentration to the next.**

We were surprised by this phenomenon as well. However, it appears to be typical for threshold determinations by the descending method of limit for CD-1 mice (see data below from Joshi et al, 2009 above which mimic our findings). The dearth of intermediate % correct responses may, in part, be an artifact of our criterion for threshold. For example, Figure 1B shows an individual mouse’s path to threshold. Note that she failed at 10^-13^ ppm but clearing was correctly making the discrimination in some blocks. However, our criterion only takes into account the last three blocks. Almost any criterion you could invent would suffer from similar issues of arbitrariness. Another factor that may explain this phenomenon is that measurement precisions is set at a full log unit!. If we had used a finer grain resolution (this could have taken much of the rest of our lives!) we may have seen more cases of intermediate-level performance near threshold. Finally, a mouse’s motivation is unstable---for a time they seem to be highly motivated by their successes when discriminations are easy but as the discriminations become more difficult they often “throw in the towel” or switch to a strategy of responding on every trial. Such are the challenges of threshold determinations in non-human animals and suggest that our thresholds, though astoundingly low, may still be underestimates of a mouse true olfactory capabilities.

**Reviewer #2:** Blount and Coppola have provided a rigorous, well-reasoned, and scholarly study, with two major results: 1) "odor enrichment" or "induction", though it causes various changes in the olfactory epithelium in bulb, and has been shown by others to affect thresholds, does not induce a detectable change in thresholds when measured thoroughly in well-trained mice. 2) well-trained mice can have remarkably low thresholds, not just for odor detection but for discrimination of enantiomers. I find result 1 convincing, and in my view this result alone provides sufficient reason to accept this study for publication. However, I have misgivings about result 2.

Water restricted mice are devious experimental subjects (not that I blame them). The authors have convincingly demonstrated that their mice were not using non-odor cues to guide their choices. However, I'm not sure the authors have eliminated the possibility that residual odor cues could be contributing to the behavioral performance they observe. In my lab, we have found in several assays that mice can perform in olfactory tasks even when presented with nominally blank vials. After all too much troubleshooting, we found that the problem was residual odor -- this performance would drop to chance after a thorough cleaning of the olfactometer. The authors performed separate "0-0" control sessions, but the problem is that residual odor can accumulate sporadically over sessions. Some days the residuum may have accumulated to a detectable level, other days not. This may explain some of the across-mouse and across-day variability in performance. In my view, the best way to properly control for this possibility is to interleave "0-0" trials into every session (see eg, Findley et al, biorXiv 2020). Dewan et al (2018) had to go to great lengths to avoid this effect (see Methods (incidentally, this study also found extremely low thresholds, and may be worth including in figure 7)) -- no-odor trials were presented from multiple blank vials, some of which treated as S+ others as S-. Importantly, mice were evaluated for "cheating" in every behavioral session.

My paranoia may be misplaced in the case of this study. I am not familiar with the Knosys olfactometer design, and it may have features that eliminate this possibility. It would be helpful to include a schematic of the design so that this can be evaluated. Otherwise, I encourage the authors to discuss whether or not an extraneous olfactory cue that varies from day to day can be excluded as a contributor to the remarkably low thresholds they observe for limonene.

**Responses to Dr. Smear’s Comments:**

We agree with Dr. Smear that water restricted mice are “devious” indeed and will use the most salient clue---olfactory or otherwise---to solve discrimination problems we pose to them. It is clear from Dr. Smear’s comment that we did not do an adequate job of explaining our remedy for this problem and have rewritten this part of the manuscript to make our methods more intelligible (p. 10-11). In practice, we operated exactly as Dr. Smear recommended in his review of our manuscript: all sessions involving low concentrations interleaved blank vs blank or S- vs S- control trials. We simply chose not to show every control trial of every mouse in the figures. Rather we gave examples of the drop off in performance in 6 mice discriminating S+ from S- trials at very low odor concentrations. However, we avoided using control trials too frequently because they cause extinction. This is a particular problem at low concentrations when the mouse is making very difficult discriminations. Thus, we did not interleave control trials in every block though they were a part of **every** session. Importantly, in our protocol, a mouse was **required** to fail the control trials before they could move on to the next concentration step.

On the issue of extraneous odors, also a key point rightly brought up by Dr. Smear, we minimized these with a thorough regimen of cleaning the olfactometer and replacement of tubing that was down-stream from the odor saturator bottles. Without such procedures, we agree, that contamination can dominate a mouse’s responding. Indeed, on the occasions where a subject failed the Blank vs Blank control blocks (they still recognized S+), simply cleaning the apparatus and changing the tubing invariably brought the mouse’s behavior back under our control. It is also worth noting that the precipitous drop in % correct responding at threshold that typify our threshold data is inconsistent with odor contamination as a confounding factor.

After careful consideration we chose not to add a schematic of the Knosys system. First, Knosys has been the most widely used system for rodent olfactometry for many years (see our references). And a schematic of the system has been published on a number of occasions (e.g. Bodyak & Slotnick, 1999; Slotnick & Restrepo, 2006; Laska, 2017). Also, though a lesser consideration, we would not want to give the impression that we had any hand in the invention or modification of the Knosys olfactometer which is the brainchild of our colleague and erstwhile collaborator Dr. Burt Slotnick.

Finally, we chose not to discuss Dewan et al., 2018 or show their data (though we were aware of this important work) because: (1) it deals with allomones which seem to us to be a very different type of stimulus than the “normal” odorants we used and (2) while low, their thresholds are several log units higher than ours and thus not particularly exemplary in the context of our paper.
